# Culturally Relevant Physical Activity in the Behavioral Risk Factor Surveillance System in Hawai‘i

**DOI:** 10.5888/pcd20.220412

**Published:** 2023-05-25

**Authors:** Tetine Sentell, Yan Yan Wu, Mele Look, Kapuaola Gellert, Tonya Lowery St. John, Lance Ching, Riko Lee, Catherine Pirkle

**Affiliations:** 1 Office of Public Health Studies, Thompson School of Social Work and Public Health, University of Hawai‘i at Mānoa, Honolulu; 2 John A. Burns School of Medicine, Department of Native Hawaiian Health, University of Hawai‘i, Honolulu; 3 Surveillance, Evaluation and Epidemiology Office, Chronic Disease Prevention and Health Promotion Division, Hawai‘i State Department of Health, Honolulu

## Abstract

**Introduction:**

Culturally relevant physical activity is a promising field for chronic disease prevention and management. Native Hawaiians and Other Pacific Islanders have higher rates of physical inactivity than other racial or ethnic groups and increased risk of chronic disease. The study objective was to provide population-level data from Hawai‘i on lifetime experiences in the Native Hawaiian Indigenous practices of hula and outrigger canoe paddling across demographic and health factors to identify opportunities for public health intervention, engagement, and surveillance.

**Methods:**

Questions about hula and paddling were added to the Hawai‘i 2018 and 2019 Behavioral Risk Factor Surveillance System (N = 13,548). We considered level of engagement by demographic categories and health status indicators, accounting for the complex survey design.

**Results:**

Overall, 24.5% of adults engaged in hula and 19.8% in paddling in their lifetime. Prevalence of engagement was higher among Native Hawaiians (48.8% hula, 41.5% paddling) and Other Pacific Islanders (35.3% hula, 31.1% paddling) than among other racial and ethnic groups. In adjusted rate ratios, experience in these activities was strong across age groups, education, sex, and income levels, particularly among Native Hawaiians and Other Pacific Islanders.

**Conclusion:**

Throughout Hawaiʻi, hula and outrigger canoe paddling are important and popular cultural practices with high physical activity demands. Participation was notably high for Native Hawaiians and Other Pacific Islanders. Surveillance information around culturally relevant physical activities can benefit public health programming and research from a strength-based community perspective.

**Summary**

**What is already known on this topic?**

Culturally relevant physical activity is a promising field for chronic disease prevention and management that can also address health inequities. However, the scale of engagement and interest in these practices are often hidden because such practices are often unmeasured by population-level surveillance. Hula dancing (hula) and outrigger canoe paddling are 2 understudied, culturally relevant physical activities in Hawai‘i.

**What is added by this report?**

This study provides detailed, population-level information on the high prevalence of hula and outrigger canoe paddling in Hawai‘i across demographic and health factors.

**What are the implications for public health practice?**

Results from this study support surveillance and promotion of culturally relevant physical activity to address chronic disease disparities from a strength-based public health perspective.

## Introduction

Only 21% of adults in the US (1) and 24.6% of adults in Hawai‘i (2) meet the US Department of Health and Human Services’ physical activity guidelines (3). Native Hawaiians and Other Pacific Islanders have higher rates of physical inactivity than other racial and ethnic groups in US population-level surveillance (4); such inactivity is associated with premature death, the development of chronic diseases (5), and increased risk of multiple morbidities (6).

Culturally relevant physical activity is exercise that is based on a population’s cultural customs and is a promising field for chronic disease prevention and management. Promoting activities of known importance in communities is a particularly meaningful method to increase collective well-being and address health inequities (7). Despite the value of culturally relevant physical activity, these practices are not typically measured by population-level surveillance. Thus, the scale of engagement and interest that could be leveraged for strength-based public health interventions is often hidden.

Hula and outrigger canoe paddling (paddling) are Indigenous practices of cultural relevance to Native Hawaiians and Other Pacific Islanders that can demand substantial levels of physical activity (8–10). Both practices are understudied, but research on paddling is especially scarce. Existing research has established that hula provides moderate-to-vigorous physical activity (8,9), can significantly lower blood pressure (11), and has additional physical, social, and spiritual benefits (7,12).

The objective of this study was to use the Behavioral Risk Factor Surveillance System (BRFSS) to quantify the statewide population-level prevalence of lifetime engagement in hula and paddling and determine associated demographic and health factors. This information can improve surveillance data on physical activity especially relevant for Native Hawaiians and Other Pacific Islanders, identify opportunities for strengths-based health promotion, highlight areas for more detailed research on culturally relevant physical activity, and inform culturally relevant physical activity surveillance in other locations. We hypothesized the prevalence of engagement in these activities would be high across demographic and health factors, especially among Native Hawaiians and Other Pacific Islanders.

## Methods

### Sample

We obtained our sample (N = 15,584 overall; 7,901 in 2018, and 7,683 in 2019) from the 2018 and 2019 Hawai‘i BRFSS (H-BRFSS). The BRFSS is a representative, gold-standard telephone-based public health surveillance system jointly coordinated by the Centers for Disease Control and Prevention and the states and territories. The main survey has core questions focusing on critical public health issues as well as optional models. States must ask the core questions to provide harmonized surveillance information and may also elect to use optional modules. Individual states may also supplement the core survey with additional questions of their own design for population-level assessment, allowing flexibility for state health priorities.

### Variables

#### Outcomes

We added 2 questions to the H-BRFSS: 1) “Over your lifetime, how much have you participated in hula, including during school, with friends and family, or in a halau (school)?” and 2) “Over your lifetime, how much have you participated in outrigger canoe paddling, including during school, with friends and family, or as part of a club?” Response choices were 1 (never), 2 (almost never), 3 (sometimes), 4 (often), 5 (very often), 7 (don’t know/not sure) and 9 (refused). We considered reports of “don’t know/not sure” or “refused” for both hula and paddling (n = 2,036) as missing and excluded them from analyses. We considered lifetime engagement for each activity as a dichotomous variable (yes = sometimes, often, or very often vs no = never or almost never).

#### Predictors

We selected demographic and health variables relevant to practical public health decision-making and prioritization. The category “race and ethnicity” followed Hawai‘i State Department of Health standards to consider heterogeneous groups of relevance to the state (13) and included Native Hawaiians, Other Pacific Islanders, Filipino, Japanese, Chinese, White, and Other race/ethnicity. For race and ethnicity, we combined missing information (n =181) with “Other.” The BRFSS survey collected data on the following demographic characteristics: age group (18–24, 25–34, 35–44, 45–54, 55–64, or ≥65 y), sex (female or male), location of residence (O‘ahu or neighbor islands), education (less than high school graduate; high school/some college; college degree), annual household income (<$15,000, $15,000–$24,999, $25,000–$49,999, $50,000–$74,999, $75,000–$124,999, ≥$125,000), marital status (married/unmarried couple or single), and employment (employed, not employed, retired, student/homemaker, or unable to work) by level of engagement in each type of activity. Health status variables, all self-reported, were self-rated health (excellent, very good, good, fair, or poor), body mass index (BMI; normal, underweight, overweight, obese) calculated according to a participant’s reported height and weight, high blood pressure (no, yes, borderline, when pregnant), current health insurance (yes or no), ever smoked 100 cigarettes (yes or no), difficulty walking or climbing stairs (yes or no), difficulty dressing or bathing (yes or no), recent medical checkup (yes or no), self-reported ever diagnosed diabetes status (none, diabetes, gestational diabetes, prediabetes), depression (yes or no), and heart disease (yes or no). We also examined whether participants met physical activity guidelines, data that were available only in the 2019 survey.

### Analyses

After removing missing data on hula or paddling, the analytic sample was 13,548. The analysis focused on understanding associations between culturally relevant physical activity and demographic and health factors. We first considered descriptive statistics of prevalence and 95% CIs for all predictors for the whole sample and among people who identified as Native Hawaiians and Other Pacific Islanders (not including Native Hawaiians). Lastly, we performed bivariate and multivariable Poisson regression models predicting the prevalence ratios (PRs) and 95% CIs of engagement in hula, and, separately, paddling, considering demographic factors. We used Poisson regression models for the PRs because odds ratios can substantially overestimate common outcomes (when prevalence > 10%) (14). Finally, we considered overlap between the 2 activities.

We used R version 4.0.5 (R Foundation for Statistical Computing) to analyze data. All analyses accounted for the complex survey design. We assigned equal weights to 2018 and 2019 survey years. The University of Hawai‘i Institutional Review Board determined this data exempt from human subjects review. Following data rules, we did not report any findings from data with an unweighted denominator less than 50 or a relative standard error <.03, because these estimates are not precise. We used a 5% α for significance.

## Results

Of the 13,548 participants in the 2018 and 2019 H-BRFSS, 2,461 were Native Hawaiian and 655 were Other Pacific Islanders (Table 1). Of all participants, 24.5% had engaged in hula and 19.8% in paddling during their lifetime (Table 2). The prevalence of engagement in hula and paddling was higher among Native Hawaiians (hula 48.8%, paddling 41.5%) and Other Pacific Islanders (hula 35.3%, paddling 31.1%) than among the overall sample.

**Table 1:** Demographic and Health Characteristics of Participants in the 2018 and 2019 Hawai‘i Behavioral Risk Factor Surveillance System

Characteristic	No. (weighted %)		
	All (N = 13,548)	Native Hawaiian (n = 2,461)	Other Pacific Islanders (n = 655)
**Sex**			
Female	7,030 (50.5)	1,343 (51.5)	342 (50.0)
Male	6,508 (49.5)	1,116 (48.5)	313 (50.0)
**Age group, y**			
18–24	795 (10.2)	210 (14.7)	86 (19.4)
25–34	1,450 (16.7)	349 (20.7)	131 (31.7)
35–44	1,707 (16.3)	394 (19.4)	130 (19.5)
45–54	1,959 (14.7)	413 (15.9)	121 (13.1)
55–64	2,723 (17.1)	477 (13.7)	83 (8.0)
≥65	4,779 (24.9)	599 (15.7)	93 (8.3)
**Education**			
Less than high school graduate	529 (8.2)	135 (10.5)	55 (13.4)
High school or some college	7,157 (61.5)	1,695 (72.3)	461 (73.5)
College degree	5,834 (30.4)	628 (17.2)	139 (13.0)
**Annual household income, $**			
<15,000	953 (6.8)	244 (10.5)	90 (14.9)
15,000–24,999	1,752 (13.9)	413 (18.2)	120 (19.1)
25,000–49,999	2,746 (21.4)	560 (22.7)	148 (27.9)
50,000–74,999	1,990 (15.9)	346 (15.1)	73 (10.8)
75,000–124,999	2,652 (23.1)	402 (19.7)	86 (15.7)
≥125,000	2,044 (18.9)	281 (13.9)	68 (11.6)
**Marital status**			
Married or partnered	7,333 (57.0)	1,219 (50.8)	339 (50.3)
Single	6,159 (43.0)	1,235 (49.2)	315 (49.7)
**Employment**			
Employed	7,561 (61.5)	1,466 (63.3)	412 (67.8)
Not employed	537 (4.6)	120 (6.4)	55 (9.8)
Retired	4,010 (22.5)	525 (15.2)	88 (9.0)
Student or homemaker	745 (7.7)	162 (9.6)	50 (8.5)
Unable to work	641 (3.8)	177 (5.4)	46 (5.0)
**Island of residence**			
O**‘**ahu	6,617 (68.4)	1,132 (61.7)	389 (75.2)
Neighbor islands	6,910 (31.6)	1,327 (38.3)	266 (24.8)
**Self-rated health**			
Excellent	2,333 (17.7)	304 (14.3)	99 (15.7)
Very good	4,292 (30.7)	594 (24.2)	130 (18.5)
Good	4,612 (35.2)	954 (38.2)	265 (41.9)
Fair	1,816 (13.1)	477 (18.9)	134 (20.1)
Poor	488 (3.3)	132 (4.3)	27 (3.8)
**Meet physical activity guidelines (2019 wave only)**			
Did not meet	2,578 (43.8)	498 (44.8)	133 (46.3)
Met	3,839 (56.2)	643 (55.2)	171 (53.7)
**Body mass index^a^**			
Normal (18.5–24.9)	5,102 (38.2)	565 (23.0)	127 (19.6)
Underweight (<18.5)	365 (2.8)	39 (1.8)	8 (2.0)
Overweight (25.0–29.9)	4,446 (33.6)	758 (30.5)	205 (31.3)
Obese (≥30.0)	3,304 (25.4)	1,042 (44.8)	297 (47.1)
**Diabetes status**			
No	9,576 (71.9)	1,572 (68.7)	436 (71.1)
Diabetes	1,708 (11.6)	431 (14.2)	112 (14.4)
Gestational diabetes	207 (1.8)	61 (2.5)	23 (3.9)
Prediabetes	2,050 (14.6)	396 (14.6)	83 (10.6)
**Hypertension**			
No	8,266 (64.8)	1,384 (64.4)	416 (70.9)
Yes	4,777 (32.7)	984 (33.9)	210 (26.5)
Borderline	278 (1.9)	37 (1.1)	13 (1.7)
When pregnant	86 (0.5)	23 (0.7)	5 (0.9)
**Depressive disorder**			
No	11,548 (87.0)	2,074 (84.7)	564 (87.9)
Yes	1,963 (13.0)	380 (15.3)	91 (12.1)
**Heart disease**			
No	12,907 (97.0)	2,313 (97.0)	619 (95.5)
Yes	547 (3.0)	120 (3.0)	27 (4.5)
**Health insurance**			
No	789 (6.3)	149 (6.9)	96 (15.3)
Yes	12,733 (93.7)	2,304 (93.1)	557 (84.7)
**Ever smoked 100 cigarettes**			
No	7,799 (61.0)	1,271 (52.7)	382 (57.9)
Yes	5,702 (39.0)	1,184 (47.3)	268 (42.1)
**Difficulty walking or climbing stairs**			
No	11,733 (89.0)	2,069 (88.7)	558 (88.8)
Yes	1,795 (11.0)	389 (11.3)	95 (11.2)
**Difficulty dressing or bathing**			
No	11,675 (97.3)	2,057 (97.0)	555 (96.7)
Yes	395 (2.7)	83 (3.0)	24 (3.3)

a Calculated as weight in kilograms divided by height in meters squared.

**Table 2:** Prevalence of Hula (Often and Sometimes) and Paddling (Often and Sometimes), by Demographic and Health Characteristics, Among Participants in the 2018 and 2019 Hawai‘i Behavioral Risk Factor Surveillance System

Characteristic	Hula, % (95% CI^a^)			Paddling, % (95% CI^a^)		
	All	Native Hawaiians	Other Pacific Islanders	All	Native Hawaiians	Other Pacific Islanders
**Overall**	24.5 (23.5–25.5)	48.8 (46.1–51.5)	35.3 (30.5–40.4)	19.8 (18.9–20.8)	41.5 (38.9–44.2)	31.1 (26.4–36.1)
**Sex**						
Female [Reference]	35.3 (33.8–36.9)	65.5 (61.9–68.9)	44.4 (37.5–51.6)	17.2 (16.0–18.5)	37.8 (34.2–41.6)	28.6 (22.0–36.2)
Male	13.5 (12.4–14.7)^b^	31.0 (27.5–34.8)^b^	26.1 (20.1–33.2)^b^	22.5 (21.2–23.9)^b^	45.4 (41.6–49.2)^b^	33.6 (27.2–40.6)
**Age group, y**						
18–24 [Reference]	35.9 (32.0–40.1)	56.8 (48.2–65.1)	44.9 (32.7–57.8)	27.1 (23.5–31.1)	41.8 (33.6–50.5)	35.0 (23.5–48.6)
25–34	30.1 (27.3–33.1)^b^	57.0 (50.4–63.3)	40.6 (30.5–51.5)	25.6 (22.9–28.5)	43.8 (37.4–50.3)	40.2 (30.0–51.2)
35–44	26.7 (24.1–29.6)^b^	49.1 (42.6–55.7)	34.6 (25.5–44.9)	22.7 (20.2–25.4)	45.0 (38.5–51.7)	23.3 (15.8–32.9)
45–54	22.9 (20.7–25.2)^b^	44.4 (38.6–50.2)^b^	25.1 (16.6–36.0)^b^	24.1 (21.8–26.5)	49.6 (43.7–55.5)	22.3 (14.9–32.0)
55–64	19.3 (17.5–21.3)^b^	45.2 (39.6–50.9)^b^	22.5 (13.9–34.2)^b^	17.1 (15.4–18.9)^b^	38.8 (33.5–44.5)	34.0 (23.1–46.9)
≥65	19.0 (17.5–20.5)^b^	38.2 (33.3–43.4)^b^	26.8 (17.0–39.7)^b^	10.7 (9.6–11.9)^b^	27.8 (23.2–32.8)^b^	17.5 (10.2–28.3)^b^
**Education**						
Less than high school graduate [Reference]	21.9 (17.5–27.1)	43.8 (32.5–55.7)	30.1 (17.2–47.1)	17.6 (13.6–22.4)	32.1 (22.0–44.1)	25.2 (13.3–42.5)
High school graduate or some college	25.8 (24.5–27.2)	48.2 (45.1–51.3)	35.9 (30.3–41.9)	21.3 (20.1–22.6)	43.7 (40.7–46.8)	32.1 (26.6–38.1)
College degree	22.6 (21.2–24.0)	54.7 (49.9–59.5)	37.1 (27.3–48.0)	17.4 (16.2–18.7)	38.1 (33.6–42.9)	31.4 (22.4–42.2)
**Annual household income, $**						
<15,000 [Reference]	27.9 (23.8–32.4)	45.1 (35.4–55.1)	35.5 (23.0–50.3)	20.8 (17.1–25.1)	34.6 (25.5–44.9)	27.6 (16.1–43.0)
15,000–24,999	29.0 (26.1–32.0)	54.4 (47.7–60.9)	34.6 (24.3–46.7)	21.1 (18.5–23.9)	40.0 (33.5–46.8)	28.9 (19.3–40.9)
25,000–49,999	26.4 (24.0–28.8)	50.2 (44.8–55.7)	41.7 (31.0–53.1)	19.2 (17.1–21.5)	42.6 (37.3–48.0)	34.9 (24.7–46.6)
50,000–74,999	26.4 (23.8–29.1)	58.0 (51.1–64.6)^b^	33.7 (21.7–48.3)	20.3 (18.0–22.8)	43.3 (36.4–50.4)	33.8 (21.7–48.6)
75,000–124,999	21.2 (19.3–23.3)^b^	47.0 (40.7–53.3)	25.8 (16.7–37.7)	19.6 (17.7–21.6)	46.6 (40.4–53.0)	26.8 (16.9–39.9)
≥125,000	19.7 (17.5–22.1)^b^	43.6 (36.5–51.1)	33.3 (19.7–50.3)	21.1 (18.9–23.6)	40.1 (33.0–47.7)	36.3 (22.3–53.1)
**Marital status**						
Married or partnered [Reference]	23.1 (21.8–24.5)	49.5 (45.9–53.1)	33.3 (27.1–40.1)	19.1 (17.9–20.3)	43.5 (39.9–47.2)	28.7 (22.9–35.4)
Single	26.4 (24.9–28.0)^b^	48.1 (44.1–52.0)	37.4 (30.3–45.0)	20.7 (19.3–22.2)	39.5 (35.7–43.4)	33.5 (26.6–41.3)
**Employment**						
Employed [Reference]	24.5 (23.2–25.8)	51.4 (48.1–54.6)	34.1 (28.2–40.5)	22.1 (20.9–23.3)	44.2 (41.0–47.4)	31.4 (25.6–37.8)
Not employed	30.2 (25.0–36.0)^b^	45.7 (32.8–59.2)	33.4 (19.7–50.7)	22.6 (18.1–27.8)	31.1 (21.3–42.9)	36.7 (22.0–54.4)
Retired	19.1 (17.5–20.9)^b^	38.0 (32.8–43.5)^b^	27.9 (18.0–40.7)	11.2 (9.9–12.5)^b^	31.7 (26.7–37.1)^b^	23.7 (14.4–36.6)
Student or homemaker	35.8 (31.4–40.5)^b^	57.9 (46.1–68.9)	51.8 (34.9–68.4)^b^	25.3 (21.1–29.9)	48.2 (36.8–59.8)	40.8 (24.4–59.4)
Unable to work	26.0 (21.6–30.9)	38.2 (29.6–47.7)^b^	41.9 (23.8–62.5)	21.0 (17.1–25.6)	38.5 (29.9–47.9)	—c
**Island of residence**						
O**‘**ahu [Reference]	23.8 (22.6–25.1)	47.8 (44.1–51.4)	36.5 (30.6–42.8)	18.0 (16.8–19.2)	39.5 (35.9–43.1)	30.5 (24.8–36.8)
Neighbor islands	25.9 (24.5–27.4)^b^	50.4 (46.6–54.3)	31.7 (25.1–39.2)	23.9 (22.5–25.3)^b^	44.8 (41.0–48.7)^b^	32.9 (25.9–40.7)
**Self-rated health**						
Excellent [Reference]	22.6 (20.4–25.1)	47.0 (39.8–54.4)	43.6 (31.1–57.0)	20.8 (18.7–23.2)	47.9 (40.7–55.3)	34.5 (22.9–48.2)
Very good	24.4 (22.8–26.2)	48.9 (43.4–54.4)	25.3 (17.4–35.2)^b^	18.7 (17.2–20.4)	41.3 (35.9–46.9)	25.9 (17.7–36.2)
Good	23.9 (22.2–25.6)	48.5 (44.2–52.7)	33.6 (26.4–41.7)	19.5 (18.0–21.2)	40.1 (36.0–44.4)	31.7 (24.4–39.9)
Fair	28.4 (25.6–31.3)^b^	51.6 (45.4–57.8)	38.6 (29.0–49.1)	21.0 (18.5–23.7)	40.5 (34.6–46.7)	28.0 (19.6–38.4)
Poor	26.6 (21.1–32.9)	45.0 (34.2–56.4)	—c	23.5 (18.4–29.6)	38.1 (28.1–49.2)	—c
**Meet physical activity guidelines (2019 data only)**						
Did not meet [Reference]	23.2 (21.1–25.3)	47.4 (41.7–53.0)	39.5 (28.8–51.2)	15.4 (13.7–17.4)	35.0 (29.8–40.5)	33.3 (22.8–45.8)
Met	26.9 (24.9–28.9)^b^	51.5 (46.5–56.6)	35.2 (27.0–44.5)	23.6 (21.8–25.6)^b^	45.3 (40.3–50.4)^b^	34.0 (25.6–43.4)
**Body mass index^d^**						
Normal (18.5–24.9) [Reference]	23.3 (21.7–25.0)	47.2 (41.6–52.9)	36.9 (25.8–49.5)	16.6 (15.2–18.0)	39.9 (34.3–45.7)	32.1 (21.4–45.1)
Underweight (<18.5)	26.9 (21.2–33.5)	54.0 (34.8–72.1)	—c	16.2 (11.6–22.2)	31.8 (16.2–52.8)	—c
Overweight (25.0–29.9)	21.7 (20.1–23.4)	48.7 (43.8–53.6)	31.9 (23.6–41.5)	19.6 (18.1–21.3)^b^	42.5 (37.7–47.4)	27.6 (19.9–36.9)
Obese (≥30.0)	29.1 (27.1–31.3)^b^	48.6 (44.6–52.7)	38.7 (31.7–46.1)	25.8 (23.8–27.9)^b^	42.8 (38.8–46.8)	32.1 (25.6–39.4)
**Diabetes status**						
No [Reference]	24.4 (23.2–25.6)	49.1 (45.7–52.5)	34.7 (29.0–40.9)	20.5 (19.4–21.6)	43.6 (40.2–47.0)	31.0 (25.6–37.1)
Diabetes	22.4 (19.9–25.2)	44.1 (38.1–50.3)	35.1 (23.1–49.3)	17.6 (15.2–20.4)	35.1 (29.4–41.2)^b^	26.6 (15.5–41.8)
Gestational diabetes	42.6 (33.4–52.2)^b^	72.4 (58.4–83.1)^b^	68.2 (44.5–85.2)^b^	20.5 (14.5–28.2)	35.9 (23.2–50.8)	38.2 (18.6–62.5)
Prediabetes	24.4 (22.0–27.1)	47.9 (41.6–54.3)	27.6 (16.7–42.0)	18.4 (16.2–20.8)	39.1 (33.1–45.5)	34.4 (21.4–50.4)
**Hypertension**						
No [Reference]	25.3 (24.0–26.6)	51.2 (47.6–54.7)	36.7 (30.9–42.8)	21.3 (20.1–22.5)	44.0 (40.6–47.6)	33.7 (28.0–39.8)
Yes	23.0 (21.4–24.8)	44.6 (40.5–48.9)^b^	31.1 (22.5–41.2)	17.3 (15.8–18.8)^b^	37.8 (33.8–42.0)^b^	24.3 (16.7–34.0)
Borderline	19.8 (14.4–26.6)	48.5 (29.1–68.3)	—c	15.1 (10.3–21.6)	38.3 (20.8–59.5)	—c
When pregnant	41.5 (29.4–54.7)^b^	62.8 (35.7–83.7)	—c	23.6 (14.0–37.0)	—c	—c
**Depressive disorder**						
No [Reference]	23.6 (22.6–24.7)	47.9 (45.0–50.8)	34.2 (29.2–39.6)	19.1 (18.2–20.1)	41.2 (38.4–44.1)	30.4 (25.5–35.9)
Yes	30.3 (27.5–33.4)^b^	53.6 (46.5–60.5)	43.1 (29.5–57.7)	24.4 (21.7–27.4)^b^	42.4 (35.6–49.5)	35.7 (23.6–50.1)
**Heart disease**						
No [Reference]	24.8 (23.8–25.8)	49.4 (46.6–52.1)	36.0 (31.1–41.3)	20.0 (19.1–21.0)	41.9 (39.2–44.6)	31.7 (26.9–36.9)
Yes	15.5 (12.0–19.8)^b^	34.2 (23.9–46.2)^b^	—c	15.6 (12.2–19.7)^b^	34.9 (24.3–47.1)	—c
**Health insurance**						
No [Reference]	25.3 (21.2–29.8)	47.4 (36.4–58.7)	35.3 (24.1–48.3)	20.8 (17.4–24.7)	41.4 (31.2–52.4)	27.1 (16.9–40.5)
Yes	24.4 (23.4–25.5)	48.9 (46.2–51.7)	35.2 (30.0–40.8)	19.7 (18.8–20.7)	41.4 (38.7–44.1)	31.4 (26.3–37.0)
**Ever smoked 100 cigarettes**						
No [Reference]	24.4 (23.1–25.8)	49.3 (45.6–52.9)	32.1 (26.4–38.4)	18.5 (17.3–19.7)	42.6 (39.0–46.3)	28.3 (22.6–34.7)
Yes	24.6 (23.1–26.2)	48.2 (44.3–52.1)	40.0 (32.1–48.4)	22.0 (20.5–23.5)^b^	40.3 (36.5–44.1)	34.7 (27.2–43.1)
**Difficulty walking or climbing stairs**						
No [Reference]	24.5 (23.5–25.6)	49.4 (46.5–52.3)	36.0 (30.9–41.6)	20.5 (19.5–21.5)	42.6 (39.8–45.5)	32.1 (27.0–37.6)
Yes	24.3 (21.6–27.2)	44.5 (38.5–50.8)	29.1 (19.0–41.8)	14.7 (12.5–17.3)^b^	33.1 (27.5–39.3)^b^	23.5 (14.8–35.3)
**Difficulty dressing or bathing**						
No [Reference]	24.6 (23.5–25.6)	49.4 (46.5–52.3)	36.2 (31.0–41.8)	20.4 (19.4–21.4)	42.5 (39.6–45.4)	32.2 (27.1–37.7)
Yes	20.2 (15.4–25.9)	45.5 (32.6–59.2)	—c	18.4 (13.9–23.9)	44.0 (31.4–57.4)	—c

a Nonoverlapping 95% CIs imply a *P* value <.01; slightly overlapping 95% CIs indicate a *P* value ranging from ~.01 to .05.

b *P* < .05 for comparison with reference group in same column.

c Estimates with a relative SE (RES) >30% were suppressed per Behavioral Risk Factor Surveillance System guidelines. RSEs were calculated by dividing the SE by the estimate. The lower the RSE, the more precise the estimate, because of less variance around the mean.

d Calculated as weight in kilograms divided by height in meters squared.

**Figure 1:**
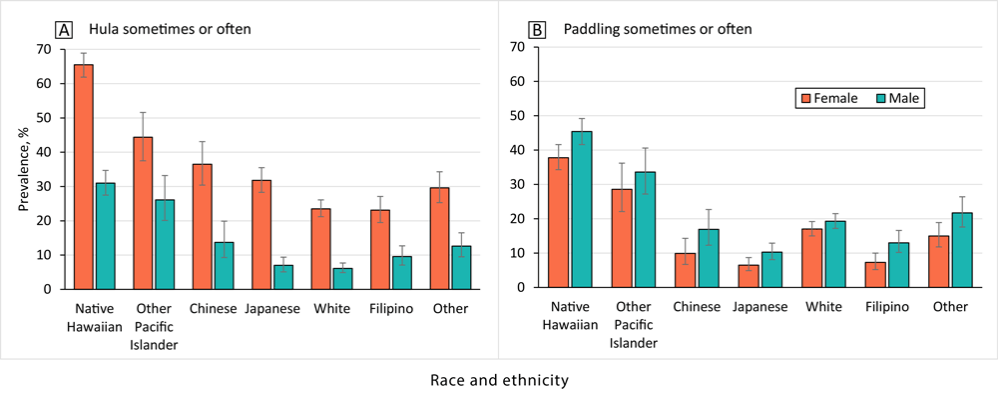
Participation in hula and paddling by sex and race and ethnicity.

The prevalence of engagement in hula and paddling was higher among Native Hawaiian and Other Pacific Islander people than among other racial and ethnic groups across sex (Figure 1). Women were more likely to have participated in hula than men (35.3% vs 13.5% overall), including 65.5% of Native Hawaiian women and 44.4% of Other Pacific Islander women, but men had also participated in hula, especially Native Hawaiian (31.0%) and Other Pacific Islander men (26.1%). Men were somewhat more likely to have participated in paddling than women. Notably, 45.4% of Native Hawaiian men and 37.8% of Native Hawaiian women had participated in paddling, as had 33.6% of Other Pacific Islander men and 28.6% of Other Pacific Islander women.

**Table:** 

Race and ethnicity	Female, % (95% CI)	Male, % (95% CI)
**Hula**		
Native Hawaiian	65.5 (61.9–68.9)	31.0 (27.5–34.7)
Other Pacific Islander	44.4 (37.5–51.6)	26.1 (20.1–33.2)
Chinese	36.5 (30.4–43.1)	13.7 (9.3–19.9)
Japanese	31.8 (28.3–35.5)	7.0 (5.1–9.4)
White	23.5 (21.2–26.1)	6.1 (4.9–7.7)
Filipino	23.1 (19.5–27.1)	9.6 (7.1–12.7)
Other	29.6 (25.3–34.3)	12.6 (9.5–16.5)
**Paddling**		
Native Hawaiian	37.8 (34.3–41.6)	45.4 (41.6–49.2)
Other Pacific Islander	28.6 (22.1–36.2)	33.6 (27.2–40.6)
Chinese	9.9 (6.7–14.3)	16.9 (12.3–22.7)
Japanese	6.5 (4.9–8.7)	10.3 (8.1–12.9)
White	17.0 (15.0–19.2)	19.3 (17.2–21.5)
Filipino	7.3 (5.2–10.0)	13.0 (10.2–16.6)
Other	15.0 (11.8–18.9)	21.7 (17.6–26.4)

### Hula

Lifetime engagement in hula did not vary significantly by education or income in any of the 3 samples (Table 2), except for those in the lowest income category (<$15,000), who had lower levels of engagement. The prevalence of hula engagement declined with age in the overall sample. We found no significant differences in lifetime engagement in hula among Native Hawaiians in groups aged 18 to 44 years. Although we found significantly lower levels of lifetime hula engagement with each increase in age group for those aged 45 to 55, 54 to 64, and 65 years or older compared with the group aged 18 to 24 years, lifetime engagement among Native Hawaiians remained high across age groups (38.2% of those ≥65 years, the age group with the lowest prevalence). We found similar patterns among Other Pacific Islanders.

Those in the overall sample who reported fair health (28.4%) were significantly more likely than those in excellent health (22.6%) to have engaged in hula (Table 2). We observed no differences in hula engagement by level of self-reported health status among Native Hawaiians. Among Other Pacific Islanders, those who reported very good health (25.3%) were less likely than those who reported excellent health (43.6%) to have engaged in hula. In the overall sample, those who met physical activity guidelines (measured in 2019 only) (26.9%) were significantly more likely to have engaged in hula than those who did not (23.2%), and those who had obesity (29.1%) were significantly more likely to have engaged in hula compared with those of normal weight (23.3%). We observed no significant differences in hula engagement by meeting physical activity guidelines or BMI categories among Native Hawaiians or Other Pacific Islanders. In all 3 groups, those who reported gestational diabetes engaged in hula at higher percentages than those with no diabetes. We found no differences for any group for health insurance, ever smoked 100 cigarettes, difficulty walking or climbing stairs, or difficulty dressing or bathing.

### Paddling

Lifetime paddling engagement declined with age (Table 2). In the overall population, the highest prevalence (27.1%) was observed among those aged 18 to 24 years, and we found significantly lower paddling engagement with each increase in age group. Among Native Hawaiians, we observed no significant differences in lifetime paddling engagement among groups aged 18 to 44 years. The group aged 65 years or older reported significantly less engagement than the group aged 18 to 24 years (27.8% vs 41.8%), but the level of engagement was still high. We found similar patterns among Other Pacific Islanders.

Lifetime engagement in paddling did not differ significantly by education, annual household income, marital status, or levels of self-reported health status in any sample. In the overall and Native Hawaiian samples, those who lived on neighbor islands were significantly more likely than those who lived on O‘ahu to have paddled (23.9% vs 18.0% overall; 44.8% vs 39.5% Native Hawaiians). We found no differences among the Other Pacific Islander sample by location of residence. In the overall and Native Hawaiian samples, those who met physical activity guidelines (measured in 2019 only) were significantly more likely than those who did not meet guidelines to have paddled (23.6% vs 15.4% overall; 45.3% vs 35.0% Native Hawaiians).

In the overall sample, those who had obesity (25.8%) were significantly more likely than those of normal weight (16.6%) to have paddled. Among Native Hawaiians and Other Pacific Islanders, we found no significant differences in paddling engagement by BMI category. We found no differences in any of the 3 samples in paddling engagement by health insurance or difficulty dressing or bathing. In the overall and Native Hawaiian samples, we found that those who had difficulty walking or climbing stairs had lower percentages of paddling engagement than those who did not.

### Adjusted prevalence ratios

#### Hula

Native Hawaiian (adjusted PR, 3.13; 95% CI, 2.79–3.52), Other Pacific Islander (adjusted PR, 2.24; 95% CI, 1.88–2.67), Japanese (adjusted PR, 1.47; 95% CI, 1.27–1.70), and Chinese (adjusted PR, 1.71; 95% CI, 1.41–2.08) respondents, and those of Other race and ethnicity (adjusted PR, 1.40; 95% CI, 1.18–1.65) were significantly more likely than White respondents to have engaged in hula (Table 3). All age groups were significantly less likely than the reference group (aged 18–24 y) to have engaged in hula: 25 to 34 years (adjusted PR, 0.86; 95% CI, 0.74–1.00); 35 to 44 years (adjusted PR, 0.79; 95% CI, 0.68–0.93); 44 to 54 years (adjusted PR, 0.70; 95% CI, 0.60–0.81); 55 to 64 years (adjusted PR, 0.62; 95% CI, 0.53–0.72); and 65 years or older (adjusted PR, 0.63; 95% CI, 0.53–0.75). Men were less likely than women (adjusted PR, 0.38; 95% CI, 0.35–0.42) and those with a college degree were more likely than those who did not complete high school (adjusted PR, 1.29; 95% CI, 1.03–1.61) to engage in hula. Household income was not significant in the model, nor was marital or employment status. Respondents on neighbor islands were significantly more likely than those on O‘ahu to have participated in hula in the adjusted model (adjusted PR, 1.10; 95% CI, 1.02–1.18).

**Table 3:** Crude and Adjusted Prevalence Ratios (PRs) for Hula (Often and Sometimes) and Paddling (Often and Sometimes), by Demographic and Health Characteristics, Among Participants in the 2018 and 2019 Hawai‘i Behavioral Risk Factor Surveillance System

Characteristic	Hula		Paddling	
	Crude PR (95% CI) [*P* value]	Adjusted PR (95% CI) [*P* value]	Crude PR (95% CI) [*P* value]	Adjusted PR (95% CI) [*P* value]
**Race and ethnicity**				
White	1 [Reference]		1 [Reference]	
Native Hawaiian	3.45 (3.08–3.86) [.001]	3.13 (2.79–3.52) [.001]	2.28 (2.05–2.53) [.001]	2.25 (2.01–2.51) [.001]
Other Pacific Islander	2.49 (2.10–2.95) [.001]	2.24 (1.88–2.67) [.001]	1.70 (1.43–2.03) [.001]	1.70 (1.42–2.04) [.001]
Filipino	1.20 (1.01–1.43) [.04]	1.12 (0.95–1.34) [.19]	0.54 (0.44–0.67) [.001]	0.58 (0.46–0.72) [.001]
Japanese	1.43 (1.24–1.66) [.001]	1.47 (1.27–1.70) [.001]	0.45 (0.37–0.55) [.001]	0.53 (0.43–0.65) [.001]
Chinese	1.81 (1.49–2.19) [.001]	1.71 (1.41–2.08) [.001]	0.73 (0.56–0.93) [.01]	0.80 (0.63–1.03) [.09]
Other	1.46 (1.23–1.73) [.001]	1.40 (1.18–1.65) [.001]	1.01 (0.85–1.21) [.88]	1.01 (0.84–1.20) [.94]
**Sex**				
Female	1 [Reference]		1 [Reference]	
Male	0.38 (0.35–0.42) [.001]	0.38 (0.35–0.42) [.001]	1.31 (1.19–1.43) [.001]	1.27 (1.16–1.39) [.001]
**Age group, y**				
18–24	1 [Reference]		1 [Reference]	
25–34	0.84 (0.72–0.97) [.02]	0.86 (0.74–1.00) [.047]	0.94 (0.79–1.13) [.52]	0.98 (0.82–1.17) [.84]
35–44	0.74 (0.64–0.87) [.001]	0.79 (0.68–0.93) [.005]	0.84 (0.70–1.00) [.052]	0.90 (0.74–1.09) [.27]
45–54	0.64 (0.55–0.74) [.001]	0.70 (0.60–0.81) [.001]	0.89 (0.75–1.05) [.16]	1.00 (0.84–1.20) [.97]
55–64	0.54 (0.46–0.63) [.001]	0.62 (0.53–0.72) [.001]	0.63 (0.53–0.75) [.001]	0.82 (0.68–0.99) [.04]
≥65	0.53 (0.46–0.61) [.001]	0.63 (0.53–0.75) [.001]	0.39 (0.33–0.47) [.001]	0.60 (0.48–0.74) [.001]
**Education**				
Less than high school graduate	1 [Reference]		1 [Reference]	
High school or some college	1.18 (0.94–1.48) [.15]	1.22 (0.98–1.50) [.07]	1.21 (0.94–1.57) [.14]	1.26 (0.98–1.62) [0.08]
College degree	1.03 (0.82–1.29) [.79]	1.29 (1.03–1.61) [.03]	0.99 (0.76–1.28) [.94]	1.26 (0.96–1.64) [.10]
**Annual household income, $**				
<15,000	1 [Reference]		1 [Reference]	
15,000–24,999	1.04 (0.86–1.25) [.69]	1.13 (0.95–1.34) [.18]	1.01 (0.80–1.28) [.91]	1.13 (0.90–1.41) [.29]
25–000–49–999	0.95 (0.79–1.13) [.54]	1.14 (0.96–1.36) [.13]	0.92 (0.74–1.15) [.49]	1.11 (0.89–1.39) [.37]
50,000–74,999	0.95 (0.79–1.14) [.56]	1.17 (0.98–1.41) [.09]	0.98 (0.78–1.22) [.83]	1.23 (0.97–1.54) [.08]
75,000–124,999	0.76 (0.64–0.91) [.003]	0.99 (0.83–1.19) [.95]	0.94 (0.76–1.17) [.59]	1.20 (0.95–1.50) [.12]
≥125,000	0.71 (0.58–0.86) [.001]	1.00 (0.81–1.22) [.98]	1.02 (0.81–1.27) [.88]	1.33 (1.04–1.70) [.02]
**Marital status**				
Married or partnered	1 [Reference]		1 [Reference]	
Single	1.14 (1.05–1.24) [.001]	0.98 (0.90–1.06) [.58]	1.09 (0.99–1.19) [.08]	1.05 (0.95–1.16) [.32]
**Employment**				
Employed	1 [Reference]		1 [Reference]	
Not employed	1.23 (1.02–1.49) [.03]	1.05 (0.87–1.26) [.62]	1.02 (0.82–1.27) [.84]	0.98 (0.78–1.23) [.88]
Retired	0.78 (0.70–0.87) [.001]	0.94 (0.82–1.08) [.42]	0.51 (0.44–0.57) [.001]	0.86 (0.73–1.01) [.07]
Student or homemaker	1.46 (1.27–1.68) [.001]	1.01 (0.88–1.16) [.89]	1.14 (0.95–1.37) [.15]	1.16 (0.97–1.38) [.10]
Unable to work	1.06 (0.88–1.28) [.54]	1.04 (0.85–1.26) [.72]	0.95 (0.77–1.17) [.65]	0.98 (0.78–1.23) [.86]
**Island**				
O**‘**ahu	1 [Reference]		1 [Reference]	
Neighbor islands	1.09 (1.01–1.18) [.04]	1.10 (1.02–1.18) [.02]	1.33 (1.22–1.45) [.001]	1.26 (1.15–1.38) [.001]

#### Paddling

Native Hawaiians (adjusted PR, 2.25; 95% CI, 2.01–2.51) and Other Pacific Islanders (adjusted PR, 1.70; 95% CI, 1.42–2.04) were significantly more likely than White respondents to have engaged in paddling, whereas Filipino (adjusted PR, 0.58; 95% CI, 0.46–0.72), Japanese (adjusted PR, 0.53; 95% CI, 0.43–0.65), and Chinese (adjusted PR, 0.80; 95% CI, 0.63–1.03) respondents were significantly less likely (Table 3). Those aged 55 to 64 years (adjusted PR, 0.82; 95% CI, 0.68–0.99) and 65 years or older (adjusted PR, 0.60; 95% CI, 0.48–0.74) were less likely than the group aged 18 to 24 years to have engaged in paddling. Men were more likely than women to have engaged in paddling (adjusted PR, 1.27; 95% CI, 1.16–1.39). We found no significant differences by education level, annual household income, marital status, or employment status. Those on neighbor islands were significantly more likely than those on O‘ahu to have engaged in paddling in the adjusted model (adjusted PR, 1.26; 95% CI, 1.15–1.38).

#### Both hula and paddling by sex

**Figure 2:**
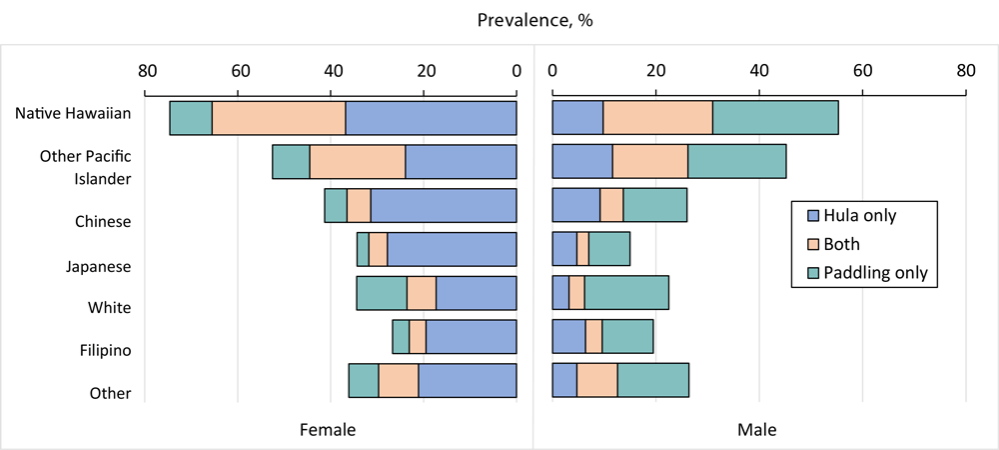
Prevalence of hula only, paddling only, and both, at least sometimes, by sex and race and ethnicity.

In the overall sample, 9.0% engaged in both hula and paddling at least sometimes, 15.5% engaged in hula only, 10.9% engaged in paddling only, and 64.6% engaged in neither at least sometimes. Among Native Hawaiians, 25.1% engaged in both at least sometimes, 23.7% engaged in hula only, 16.5% engaged in paddling only, and 34.7% engaged in neither at least sometimes. Among Other Pacific Islanders, 17.6% engaged in both at least sometimes, 17.7% engaged in hula only, 13.5% engaged in paddling only, and 51.2% engaged in neither at least sometimes. We found similar percentages of engagement in hula and paddling and both among women and men in the Native Hawaiians and Other Pacific Islanders samples (Figure 2).

**Table:** 

Race and ethnicity	Female, %			Male, %		
	Hula only	Both	Paddling only	Hula only	Both	Paddling only
Native Hawaiian	36.8	28.7	9.1	9.8	21.2	24.3
Other Pacific Islander	23.9	20.6	8.0	11.6	14.6	19.0
Chinese	31.4	5.1	4.8	9.2	4.5	12.3
Japanese	27.8	4.0	2.5	4.7	2.3	8.0
White	17.3	6.3	10.8	3.2	3.0	16.3
Filipino	19.5	3.6	3.6	6.4	3.2	9.9
Other	21.1	8.6	6.4	4.7	7.9	13.8

## Discussion

In accordance with our hypothesis, we found a high prevalence of engagement in hula and paddling among H-BRFSS respondents, especially among Native Hawaiians and Other Pacific Islanders. Almost half of all Native Hawaiians (48.8%) and more than one-third of Other Pacific Islanders (35.3%) had participated in hula, and paddling participation was 41.5% and 31.1%, respectively. Although we found decreases in older groups, engagement was strong for Native Hawaiians and Other Pacific Islanders even in older ages. Importantly, engagement with these culturally relevant physical activities was strong across education, sex, and income levels, demonstrating its broad appeal and accessibility.

These findings are important for efforts seeking to promote physical activity in Hawai‘i, especially among populations with a high risk for cardiometabolic chronic conditions. Native Hawaiians and Other Pacific Islanders are among the fastest growing racial and ethnic groups in the US (15), with some of the highest rates of diagnosed chronic diseases in the country (16). As such, it is important to identify culturally relevant and preferred physical activity options. Preventing and controlling chronic disease are key issues in Native Hawaiian health promotion, and hula is a promising means to do so (7). Among Native Hawaiians, 72.4% of those reporting gestational diabetes, 44.1% reporting diabetes, 44.6% reporting hypertension, and 34.2% reporting heart disease reported engagement in hula, suggesting relevance of this form of physical activity to this population.

Our study also suggests areas for future research and practice. Hula and paddling have some similarities and important areas of overlap, which suggest relevance of both for health promotion by sex and gender and other demographic characteristics, especially among Native Hawaiians and Other Pacific Islanders. These physical activities are also distinct, as seen in the adjusted models. Predictive factors varied, particularly by middle age and sex, which are areas to understand and consider in health promotion research and practice. Levels of engagement in hula and paddling were higher on the rural neighboring islands than on urban O‘ahu. Rural communities have a substantial burden of chronic disease and elevated rates of hypertension and diabetes (17). Identifying and promoting popular and culturally relevant physical activity options is important for these locations. Pregnant women are an important group to consider for intervention given their high levels of engagement in these activities and the prevalence of those with gestational diabetes in all 3 samples.

### Public health implications

Our work can help inform intervention design, expansion, and dissemination of culturally relevant physical activity and has critical implications for public health practice, research, population data surveillance, and location of health education delivery (18). Incorporating culture into health promotion can be an effective way to improve intervention delivery and inclusivity (19). This research points to people who would be relevant for such interventions, including Native Hawaiians, older communities, pregnant women, and middle-aged men, who can be difficult to engage in health-related interventions.

Although increasing physical activity is important, paddling and hula are more than fitness; they are healing practices for physical and emotional wellness. Their participation options are amenable to all body types and backgrounds, and participants are not expected to have a particular fitness level or be a certain age. Cultural education is an essential part of these activities; the embedded stories, values, and meanings provide a way to convey cultural wisdom and traditions. Hula and paddling organizations systematically bring Native Hawaiian values into their training. For example, *kuleana* embodies the value of responsibility to the natural world and ones’ teammates in paddling, and an expectation of *aloha* exists for everyone in the canoe because everyone is working together in an ocean that requires respect. Promoting the cultural practices of a community is a way to improve the physical activity and health of its members. To uphold the deeper meaning of these practices, it is critically important that programs promoting them engage respectfully and meaningfully with cultural practitioners for full value and benefit (7,18).

Our research has important implications for other communities and Indigenous cultural practices and highlights the importance of inclusivity in access to physical activity. The cultural relevance of these activities may add incentive for participation (20), but many people from relevant cultural backgrounds may lack time, funds, or access to participation. Reducing challenges in access and cost for these cultural practices may be fruitful areas for innovative public health and health care systems intervention (21). Teaching and integrating culturally relevant physical activities in schools may be an important way to build access, experience, and engagement equitably.

Our research points to the importance of measurement. State-level surveillance showed high levels of engagement in hula and paddling that is not captured on a typical surveillance instrument (22). The yearly BRFSS includes the following question: “During the past month, other than your regular job, did you participate in any physical activities or exercises such as running, calisthenics, golf, gardening, or walking for exercise?” The BRFSS physical activity module includes open-ended responses for the 2 most frequent physical activities in the past 30 days. Responses are then coded for national harmonization and reported in those categories, which can exclude many culturally relevant practices that may account for substantial physical activity in some communities and states (23). Systematic exclusion renders important pieces of information invisible, which was revealed in our study by the explicit question. Other states may wish to include other options in population-based surveillance for lifetime physical activities relevant to their communities.

### Limitations

This study has strengths and limitations. The questions were asked about activity during the respondents’ entire lifetime because we are not able to determine the age at which participation occurred and at what scale and intensity it occurred over time. This is an important area for future research. Older respondents were significantly less likely to report engagement in hula, which could be a form of recall bias. A Hawaiian cultural renaissance in the 1970s that is still building today has increased engagement in hula and paddling in school settings over time, which may also explain differential engagement by age cohort (24). To consider equity and timing of access and interest, future research could separate those who never participated from those who participated very little to identify how many people have at least some experience with hula or paddling. This can inform age- and sex-specific interventions.

This cross-sectional study cannot illuminate the directionality of effects, and even with this population-level data, we may lack power to detect differences in outcomes, especially in groups with small numbers of respondents. Because our study measured lifetime prevalence, its focus was not on causality but on what the H-BRFSS data revealed to us about opportunities for interventions from a strengths-based perspective. The high numbers of people who engaged in these 2 physical activities and self-reported poor health likely indicates participants being from groups who often experience health inequities, indicating an important opportunity for meaningful public health intervention.

### Conclusion

We found a high overall prevalence of lifetime engagement in hula and paddling in Hawai‘i, particularly among Native Hawaiians and Other Pacific Islanders across key demographic and health factors. Given the strong and growing evidence base in historical knowledge and intervention research on the health benefits of hula and paddling, health promotion efforts should consider encouraging and reducing barriers to participation in these activities to support health from a strengths-based and community-relevant perspective (7,8,11,12,20). Public health research and surveillance should ensure these practices are measured and studied at population levels to maximize benefits across communities.
